# High-resolution *in situ* analysis of Cas9 germline transcript distributions in gene-drive *Anopheles* mosquitoes

**DOI:** 10.1093/g3journal/jkab369

**Published:** 2021-11-15

**Authors:** Gerard Terradas, Anita Hermann, Anthony A James, William McGinnis, Ethan Bier

**Affiliations:** 1 Section of Cell and Developmental Biology, University of California, San Diego, La Jolla, CA 92093, USA; 2 Tata Institute for Genetics and Society, University of California, San Diego, La Jolla, CA 92093, USA; 3 Department of Microbiology and Molecular Genetics, University of California, Irvine, CA 92697, USA; 4 Department of Molecular Biology and Biochemistry, University of California, Irvine, CA 92697, USA

**Keywords:** *Anopheles*, *gambiae*, *stephensi*, gene drive, active genetics, CRISPR, Cas9, expression, FISH, *in situ*, *vasa*, *nanos*, microscopy

## Abstract

Gene drives are programmable genetic elements that can spread beneficial traits into wild populations to aid in vector-borne pathogen control. Two different drives have been developed for population modification of mosquito vectors. The Rec*kh* drive (*vasa*-Cas9) in *Anopheles stephensi* displays efficient allelic conversion through males but generates frequent drive-resistant mutant alleles when passed through females. In contrast, the *AgNosCd-1* drive (*nos*-Cas9) in *Anopheles gambiae* achieves almost complete allelic conversion through both genders. Here, we examined the subcellular localization of RNA transcripts in the mosquito germline. In both transgenic lines, Cas9 is strictly coexpressed with endogenous genes in stem and premeiotic cells of the testes, where both drives display highly efficient conversion. However, we observed distinct colocalization patterns for the two drives in female reproductive tissues. These studies suggest potential determinants underlying efficient drive through the female germline. We also evaluated expression patterns of alternative germline genes for future gene-drive designs.

## Introduction

Mosquitoes of the genus *Anopheles* serve as vectors for *Plasmodium* parasites, the etiological agents of malaria. Malaria is the most devastating vector-borne disease worldwide, affecting more than 225 million people yearly, especially across Sub-Saharan Africa and India (World Health Organization 2020). Classic vector control strategies, such as insecticide-treated nets or indoor residual spraying (Lengeler 2004; Pluess *et al.* 2010), have been applied successfully over the past two decades in endemic areas to reduce the disease burden by nearly 50%. Despite this progress, the downward trend in malarial cases has stalled and perhaps is reversing (World Health Organization 2020). Thus, there is a pressing need for new technologies to stay on track in the global malaria eradication agenda. One promising genetic approach is the development of species-specific CRISPR gene drives with the autonomous capacity to spread throughout native mosquito populations. Gene drives can be employed either to suppress mosquito populations (Hammond *et al.* 2016; Kyrou *et al.* 2018; Pham *et al.* 2019) or to replace disease-susceptible wild-type mosquitoes with disease-resistant individuals carrying antimalarial effectors (Adolfi *et al.* 2020; Carballar-Lejarazú *et al.* 2020). Biasing inheritance of a favorable genetic trait was conceived originally in the context of balanced translocations (Curtis 1968) and extended to selfish genetic elements to reshape the genetic makeup of wild insect populations (Burt 2003). However, practical implementation of such strategies has not been feasible until recently, following the application of CRISPR-Cas genome-editing technologies to develop self-copying gene-drives in insects (Gantz and Bier 2015). Currently, such CRISPR-based drive systems are being explored to control populations of animals that vector disease (Adolfi *et al.* 2020; Carballar-Lejarazú *et al.* 2020), impact biodiversity (Harvey-Samuel *et al.* 2017; Grunwald *et al.* 2019) or damage economically important crops (Barrett *et al.* 2019). These elements can be autonomously propagating full-drives or act on more local scales as split drives in which a Cas9 source inherited in a Mendelian fashion drops out of the population (Gantz *et al.* 2015; Li *et al.* 2020; Terradas *et al.* 2021). 

CRISPR-based drives exploit the Cas9 endonuclease and guide RNAs (gRNAs) to induce site-specific double-strand DNA breaks that either lead to desired drive-copying outcomes via homology-directed repair (HDR; Gantz and Bier 2015), or alternatively, to generation of cleavage-resistant mutant alleles via the nonhomologous end-joining (NHEJ) pathway (Unckless *et al.* 2017). NHEJ alleles, typically generated at higher frequencies in zygotic stages prior to germline allocation where allelic conversion takes place in females, can impede spread of the gene-drive system if they do not carry a detrimental fitness cost to the organism. While several second-generation drives have overcome this hurdle by different molecular mechanisms (Marshall *et al.* 2017; Champer *et al.* 2018; Adolfi *et al.* 2020; Terradas *et al.* 2021), there is a pressing need to elucidate the relationship between Cas9 endonuclease expression/activity and drive performance to inform the design of future drive systems in diverse organisms. In particular, understanding mechanisms that determine the efficiency of copying *vs* generation of resistant alleles is essential for the success and safe deployment of gene-drive technologies.

Two efficient gene drives have been developed for population modification, one targeting the major African malarial vector, *Anopheles gambiae* (Carballar-Lejarazú *et al.* 2020), and the second the Indo-Pakistani Asian vector, *Anopheles* *stephensi* (Adolfi *et al.* 2020). In laboratory cage trials, both systems drive rapidly to replace a reference nondrive population. While reproducibly achieving highly penetrant introduction of the drive element, even when seeded at low frequencies, the molecular mechanisms underlying their efficient performance differ. The *AgNosCd-1* drive in *A. gambiae* (Carballar-Lejarazú *et al.* 2020), designated herein as *cd:nos*-Cas9, is inserted in the *cardinal (cd)* locus and expresses the *Streptococcus pyogenes* Cas9 endonuclease under the control of *nanos (nos)* promoter sequences. *cd:nos*-Cas9 displays highly efficient drive when passed through both genders and generates few NHEJ alleles, resulting in the drive spreading into populations without significant accumulation of resistant alleles (Carballar-Lejarazú *et al.* 2020). The *A. stephensi* counterpart (Rec*kh*; Adolfi *et al.* 2020); designated herein as rec*kh:vas*-Cas9), inserted into the *kynurenine hydroxylase* (*kh*) locus, expresses Cas9 under the control of the *vasa* promoter and carries a recoded cDNA copy of the *kh* gene that restores endogenous gene function upon copying. This drive copies at extremely high frequencies when transmitted through males. However, in contrast to *cd:nos*-Cas9, rec*kh:vas*-Cas9 generates a significant frequency of NHEJ-induced drive-resistant events when passed through females (Pham *et al.* 2019; Adolfi *et al.* 2020). These NHEJ alleles, which are mostly loss-of-function (*kh^–^*) alleles, survive in male offspring, but carry high viability and reproductive fitness costs in females (Pham *et al.* 2019) resulting in their rapid culling from the population by a dominantly acting process designated “lethal/sterile mosaicism” (Guichard *et al.* 2019; Adolfi *et al.* 2020). These distinct drive properties, particularly evident in females, suggest that relevant differences in promoter-driven Cas9 expression patterns might contribute to the differential performance of these drives during oogenesis. In anautogenous insects (and in the gene drive field), research of developmental genes has relied mostly on quantitative PCR or fusion proteins that act as reporters for their presence in the germline, but do not accurately provide an indication of their DNA/RNA subcellular localization, which is an essential feature for proper Cas9-mediated cleavage and repair. In this study, we modified an insect multiplex fluorescence *in situ* hybridization (FISH) technique and paired it with high-resolution confocal microscopy to elucidate two primary questions important to the gene-drive field: (1) what is the optimal temporal and spatial expression of Cas9 to sustain HDR-mediated cassette copying while minimizing NHEJ outcomes in current CRISPR-based gene drives? and (2) do *cis*-regulatory promoter elements chosen to direct Cas9 expression faithfully recapitulate expression patterns of the endogenous germline gene (*vasa* or *nos*)? Another related question is whether there might be any potential species-specific differences in germline gene regulation that could underlie differential drive performance.

We addressed these questions by comparing transcript distribution patterns of Cas9 transgenes driven by the *vasa* (*A. stephensi*) or *nos* (*A. gambiae*) promoters in the germline of transgenic individuals to those of the endogenous genes. We observe overall strong concordance between promoter-driven Cas9 and endogenous gene expression patterns for both drive systems in males, but also find differences in the female germline of *A. gambiae*. In addition, we survey expression patterns of selected candidate genes using these high-resolution methods whose control sequences might be considered for future applications.

## Materials and methods

### Mosquito rearing and maintenance

*Anopheles stephensi* and *A.* *gambiae* mosquito lines were obtained from the James’ lab at University of California, Irvine and maintained at Bier’s ACL-2 insectary under standard rearing conditions of 27°C at 77% humidity and a 12 h day/night lightning cycle. Larvae were reared in distilled water and were fed a diet of powdered TetraMin^®^ fish food (Tetra, Germany). Adults were kept with 10% sucrose solution *ad libitum*. Blood meals were provided with defibrinated calf blood (Colorado Serum Co., CO, USA) and administered using a membrane-feeding system (Hemotek Ltd, UK). All handling protocols followed safety procedures approved by the University of California, San Diego.

### RNA probe design and preparation

Probes used for the study were prepared by initially amplifying genomic *A. stephensi* and *A. gambiae* DNA and PCR products were cloned into pCR™II-TOPO™ vector (Thermo Fisher Scientific, MA, USA). After successful transformation, selected bacterial colonies were isolated and plasmid templates sequenced. Haptenylated anti-sense mRNA probes were synthesized as described previously (Kosman *et al.* 2004) using the SP6 or T7 promoter, dissolved in 200 µl hybridization buffer, and stored at −20°C for later use. Probes were prepared for the following endogenous genes: *β_2_-tubulin* (1.5 kb, ASTE003208), *vasa* (1.9 kb, ASTE003241), *nos* (1 kb, AGAP006098), *oskar* (1.5 kb, ASTE003241), *snail* (1.8 kb, ASTE008611), *spo11* (2 kb, ASTE08988), *zpg* (1.3 kb, ASTE011088). The same probe targeting *Anopheles*-optimized *Cas9* was used in both species as it is a shared feature between *cd:nos* (Carballar-Lejarazú *et al.* 2020) and rec*kh:vas* (Adolfi *et al.* 2020) transgenes. Primers used for the generation of probes can be found in Supplementary Table S1.

### *In situ* hybridization and staining

Ovaries and testes of 4- to 5-day-old adult *A. stephensi* and *A. gambiae* mosquitoes were dissected into phosphate-buffered saline (PBS) solution on ice. Tissue samples were fixed within 1 h of collection for 30 min in 4% paraformaldehyde. After 3–4 washes for 5 min each in PBT (PBS + 0.1% Tween-20) to completely remove the paraformaldehyde from the solution, samples were stored in methanol at −20°C. mRNA *in situ* hybridization was carried out as described previously (Kosman *et al.* 2004) using the gene-specific probes shown above and presented in Supplementary Table S1. Samples were incubated overnight at 4°C with primary sheep anti-DIG (1:600, Sigma-Aldrich, Cat# 111333089001) and/or primary mouse anti-FITC (1:100, Sigma-Aldrich, Cat# 11426320001). The following day, after washing off the primary antibody, samples were incubated for 2 h at RT with secondary donkey anti-sheep antibodies conjugated to Alexa Fluor^®^ 488 (1:400, Thermo Fisher Scientific, Cat# A-11015) and/or donkey anti-mouse antibodies conjugated to Alexa Fluor^®^ 555 (1:400, Thermo Fisher Scientific, Cat# A-31570). Nuclei were stained with DAPI (4′,6-diamidino-2-phenylindole; Invitrogen, CA, USA), which was added to the washing buffer at a 1:500 dilution. Ovaries and testes were mounted in ProLong™ Diamond Antifade (Thermo Fisher Scientific, MA, USA) and imaged 3–10 days after mounting.

### Image acquisition and composition

The fluorescent testes and ovaries were imaged on a Leica TCS SP8X confocal microscope with white light laser and lighting super-resolution (Leica Microsystems, Germany). Either single slice or image stacks were taken. Images were deconvolved using the microscope’s built-in software (Leica Application Suite X).

### RT-qPCR and analysis

Tissues were dissected, placed directly in RNAlater™ (Thermo Fisher Scientific, MA, USA), and held for less than an hour on ice to minimize nucleic acid degradation. RNA extraction was performed using RNeasy Mini kit (QIAGEN, Germany) and the concentration obtained by NanoDrop™ One (Thermo Fisher Scientific, MA, USA). Cycles of reverse transcription were performed to obtain cDNA using SuperScript™ II Reverse Transcriptase (Thermo Fisher Scientific, MA, USA) and diluted 1:50 before its use in subsequent qPCRs. Runs were performed using iQ™ SYBR^®^ Green (Bio-Rad Laboratories Inc., CA, USA) on a MyiQ™ single color real-time PCR detection system (Bio-Rad Laboratories Inc., CA, USA). All qPCR (gene) values were normalized to the housekeeping *A. stephensi* or *A. gambiae rpS7*. Expression ratios were obtained using the ΔΔCt method (Livak and Schmittgen 2001). qPCR primers can be found in Supplementary Table S1.

### Figure generation and statistical analysis

Confocal images were processed using the Leica Application Suite X software and edited using Adobe Photoshop CC 2019 (Adobe Inc., CA, USA). All schemes and modifications of the qPCR graphs were produced using Adobe Illustrator CS6 (Adobe Inc., CA, USA). These, along with their statistics, were initially generated using Prism 8 (GraphPad Software Inc., CA, USA).

## Results

**Figure 1:**
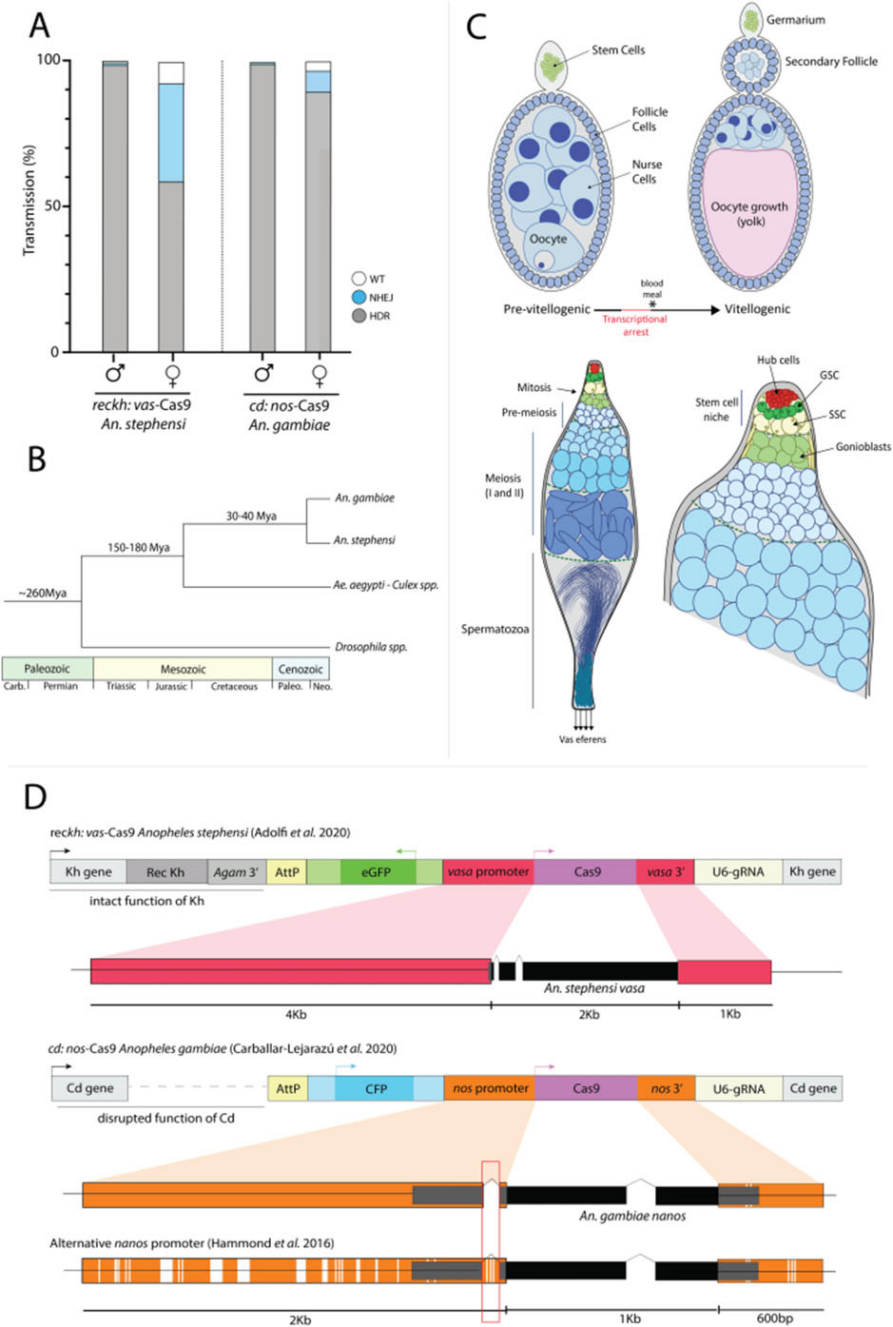
Gene-drive transmission and NHEJ rates differ for *A. stephensi* and *A. gambiae* in a sex-dependent manner. (A) Copying efficiencies of the modification gene drives: rec*kh:vas*-Cas9 (Rec*kh*) in *A. stephensi* and *cd:nos*-Cas9 (AgNosCd-1) in *A. gambiae*. Gray, blue and white bars show gene-drive system transmission, generation of NHEJ and untouched WT alleles, respectively, through males or females. (B) Phylogenetic relationships between dipteran hematophagous and nonblood sucking insects. (C) Schematics of pre/post-blood meal ovarian follicles (top) and testes (bottom). Important developmental stages and cellular types are depicted in both tissues. (D) Diagrams of *A. stephensi* and *A. gambiae* gene-drive constructs. The promoter sequences used to drive Cas9 are highlighted in red and orange, respectively; and the latter compared to an alternative construct that displays lower driving capacity. Polymorphisms between the two *nanos* promoters can be observed in white on the bottom sequence, since one study used a consensus sequence while the other used a natural variant.

As summarized earlier, the *cd:nos*-Cas9 drive is efficiently transmitted through both male and female germlines in *A. gambiae* (Carballar-Lejarazú *et al.* 2020), whereas the rec*kh:vas*-Cas9 element only drove efficiently via males in *A. stephensi* (Gantz *et al.* 2015; Adolfi *et al.* 2020; Figure  1A). We examined the distribution of Cas9 expression in the germlines of these two transgenic mosquitoes to assess whether their differential efficiencies might be related to the fidelity of promoter-driven Cas9 expression or, alternatively, to differences in endogenous gene expression (*e.g.*, *vasa*/*nos*) including possible species-specific differences in orthologous gene expression. We dissected male and female gonads (testes and ovaries, respectively) of transgenic *A. gambiae* and *A. stephensi*, hybridized them with antisense RNA probes to the products of the endogenous *nos* and *vasa* genes, and then examined multiplex gene-specific fluorescence patterns by confocal microscopy. Since the two transgenic lines evaluated here were developed in species that diverged ∼40 Mya (Figure  1B; Neafsey *et al.* 2015), we were not expecting to detect large-scale transcriptional differences or mRNA localization between the regulatory components of the well-conserved endogenous *vasa* and *nos* genes or the promoter elements used to express the Cas9 transgenes.

Spermatogenesis in male anopheline mosquitoes follows a clear spatial progression from diploid germline cells to haploid spermatozoa and is organized in a proximal–distal axis defined by the stem cell niche (proximal), located at the tip of the testes, to mature sperm exiting the germline through the *vas eferens* (distal; Figure  1C). Due to the continuous production of sperm, all developmental stages of the male germline are present in a single adult testis, which we isolated from ∼5-day-old mosquitoes in this study, unless stated otherwise. In contrast, oogenesis in anautogenous mosquitoes follows a more regulated development pathway. Mosquito eggs begin to develop after emergence, whereupon they grow to a certain stage (previtellogenic) and then arrest until a blood meal is taken (Clements and Boocock 1984). Prior to and during the arrest phase, nurse cells containing polytene chromosomes (Zhimulev and Koryakov 2009) facilitate high levels of transcription for those genes needed for oocyte and early embryonic development (Royzman *et al.* 2002). Transcripts are delivered into the transcriptionally inactive oocyte via microfilament-based contracting ring canals (Gutzeit 1986; Dana *et al.* 2005). Intake of a blood meal acts as the trigger to reactivate the transcription machinery and to complete the final stages of egg development to achieve maturation (Figure  1C). During these later stages, nurse cells begin to undergo developmentally induced apoptosis (Foley and Cooley 1998) and their remaining contents are degraded or translocated into the oocyte (Royzman *et al.* 2002). In accordance with this conditional two-stage developmental program, we dissected ovaries at two different time points (∼5 days old—pre-blood meal, ∼8 days old—24 h postblood meal). This protocol permitted examination of gene expression and mRNA localization patterns both prior to and following transcriptional arrest, as well as potential shifts in RNA localization between different cellular compartments.

### High-resolution comparison of Cas9 transcript expression and localization in anophelines

**Figure 2:**
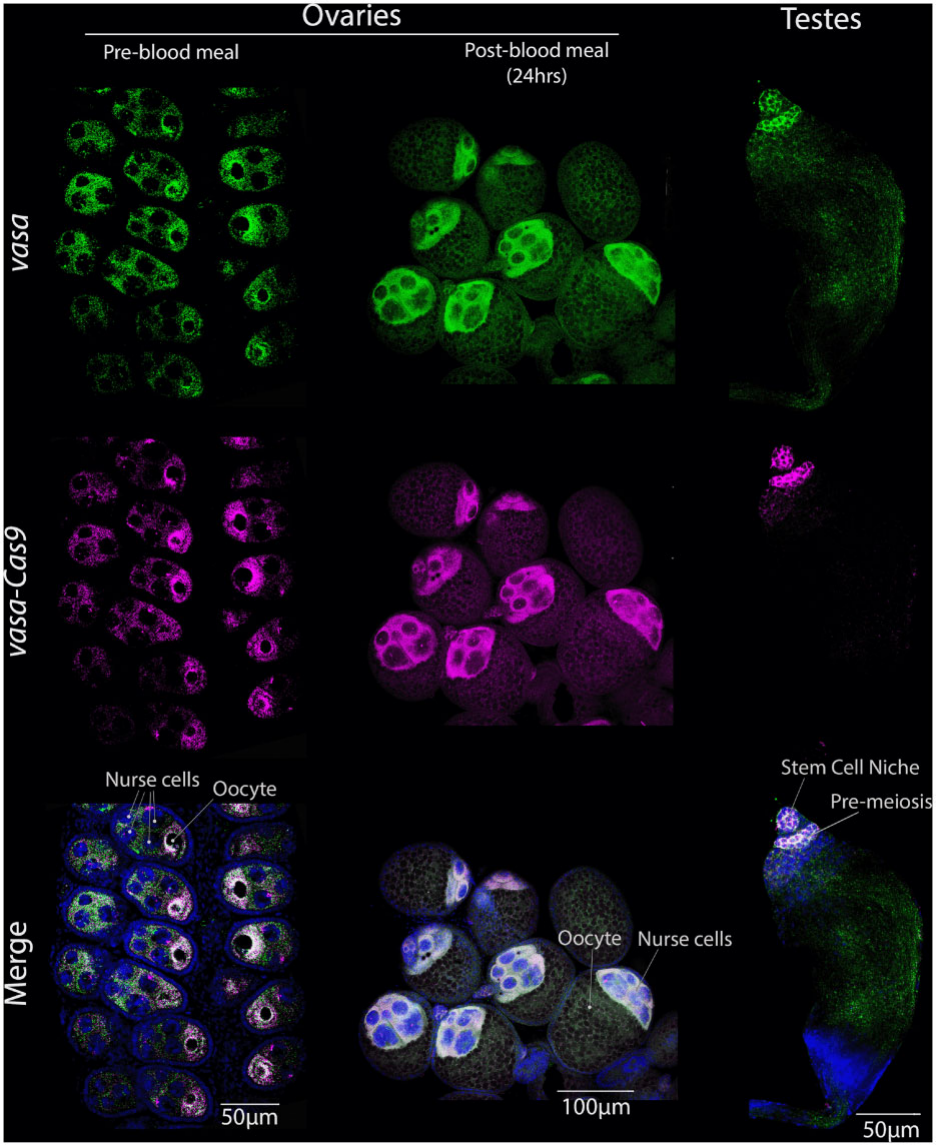
Endogenous *vasa* and *Cas9* transgene expression and transcript localization in the germline of *A. stephensi* rec*kh:vas*-Cas9 mosquitoes. Columns (left to right) depict pre or post-blood meal ovaries and testes. Rows (top to bottom) show the RNA presence of *vasa* (green), *vasa*-driven *Cas9* (magenta) and their overlap (pearl). RNA localization for both genes correlates well in ovaries, especially translocating around the oocyte pre-blood meal. In testes, *vasa* and *Cas9* RNA are seen early in development, specifically in premeiosis and germline stem cells.

We designed and synthesized RNA probes complementary to the endogenous *vasa* (*A. stephensi*) and *nos* (*A. gambiae*) genes and Cas9 transgene (identical probe for both species) and performed FISH using high-resolution confocal microscopy to examine cell-type specificity of mRNA expression as well as sub-cellular transcript localization patterns in male and female germline tissues. We first compared accumulation of the endogenous *vasa* locus and the *vasa*-driven Cas9 transgene in the *A. stephensi* rec*kh:vas*-Cas9-drive strain. We were able to detect abundant transcripts of both genes in ovaries pre- and post-bloodfeeding, as well as in the testes (Figure  2). Pre-blood meal ovaries displayed a high degree of overlap between accumulation of the endogenous *vasa* gene and *vasa*-driven Cas9 transgene transcripts surrounding the oocyte (Figure  2, Supplementary Figure S1). These concordant transcript patterns validate the identification of *vasa* promoter sequences that drive normal expression of the Cas9 transgene. In the ovary, both the C*as9* and *vasa* genes were transcribed initially in nurse cells (see independent green and magenta transcriptional dots in each nurse cell) and shortly thereafter these transcripts became localized to, and concentrated around, the oocyte. At this stage, we could distinguish the seven nurse cells from the single oocyte-fated cell based on the intensity of DAPI staining as a consequence of nurse cells being polyploid *vs* the diploid content of the oocyte nucleus, the chromosomal contents of the latter being barely detectable using our standard microscope settings (Figure  2, Supplementary Video S1). Following a blood meal, the oocyte starts growing, its nucleus doubles in size (Fiil 1974) and a shift in RNA localization occurs. While high levels of transcripts remained present in nurse cells, no further RNA import into the oocyte appeared evident. Thus, we observed strong mRNA accumulation throughout cytoplasmic regions surrounding the nurse cells (Figure  2). In the testes, there was also a tight correlation between the patterns of endogenous and *Cas9* transcript localization (Figure  2, Supplementary Figure S1). Both *vasa* and *Cas9* transcripts were present only in proximal areas of the testes distant from the *vas eferens*.

**Figure 3:**
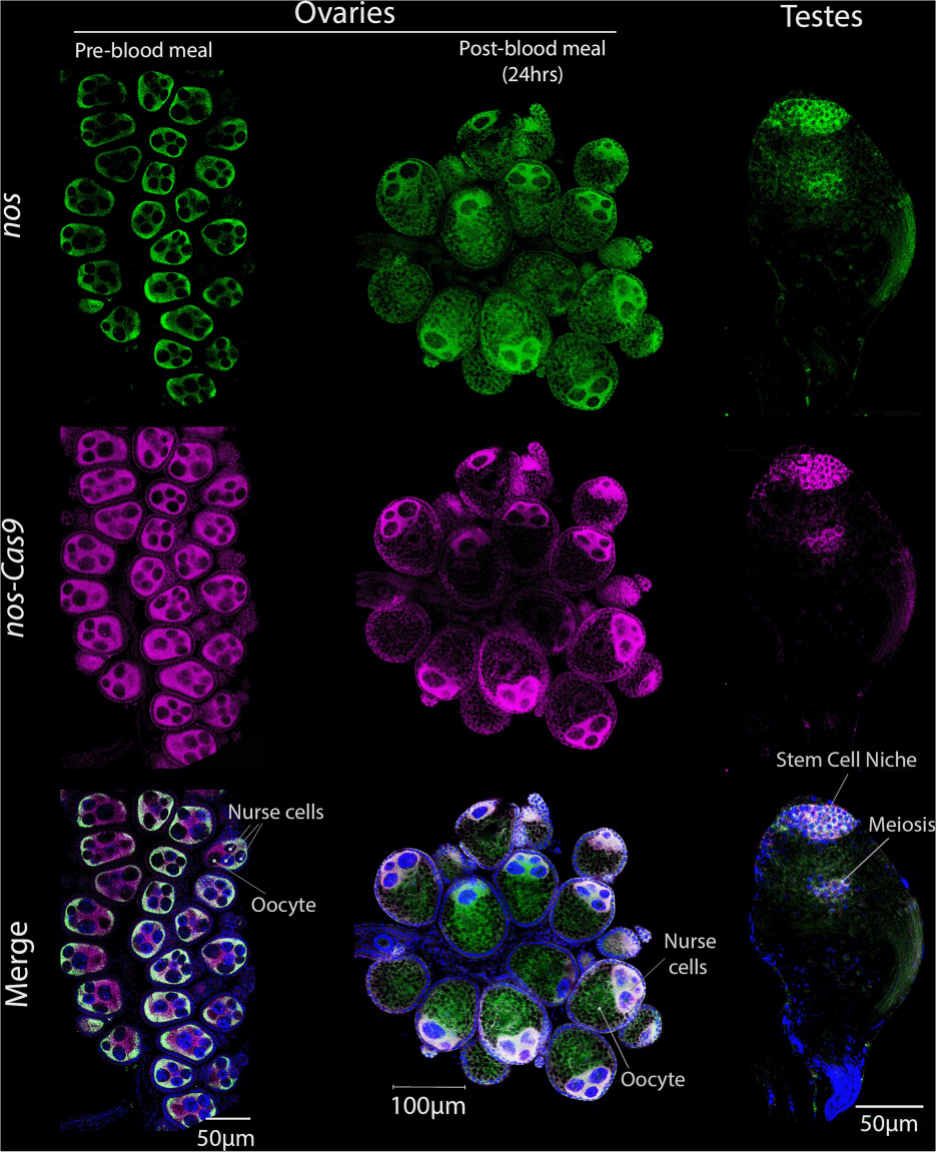
Endogenous *nos* and *Cas9* transgene expression and transcript localization in the germline of *A. gambiae cd:nos-*Cas9 mosquitoes. Columns (left to right) depict pre and post-blood meal ovaries or testes. Rows (top to bottom) show the RNA presence of endogenous *nos* (green), *nos*-driven *Cas9* (magenta) or the overlap (pearl). RNA in pre-blood meal ovaries correlates on the outer regions of the follicle, but only Cas9 is seen in the mid-section. Similarly, we observe differences post-blood meal with only nos being detectable in the developed oocyte. In testes, both genes are detected very early in development (Stem cell niche) but also slightly in cells undergoing meiosis.

The spatial patterns of *nos*-Cas9 transcripts in the germline of nonbloodfed *cd:nos*-Cas9 *A. gambiae* females paralleled that of the endogenous *nos* locus on a gross cellular level (*e.g.*, confined to nurse cells and the oocyte), but did not overlap on a subcellular scale (Figure  3, Supplementary Figure S2). This divergence in transcript localization was particularly evident throughout the cytoplasm surrounding the outer portion of the follicle, where Cas9 transcripts accumulated in the absence of a corresponding *nos* signal (Figure  3—magenta only, Supplementary Video S2). We also examined transcript localization patterns in the *A. gambiae* female germline following a blood meal. While post-bloodfed *A. stephensi* ovarian samples revealed coincident accumulation of *vasa*-Cas9 and endogenous *vasa* transcripts (Figure  2), patterns of *nos-cas9* and endogenous *nos* in *A. gambiae* transcripts displayed a notable difference. Endogenous *nos* transcripts were observed in nurse cells but also in the oocyte at lower levels, while Cas9 transcripts were restricted only to nurse cells. We also tested for differences between *nos* endogenous and *nos*-driven Cas9 transcripts by qPCR (Supplementary Figure S3). Relative to *A. gambiae rpS7*, Cas9 transcript abundance was several fold lower than for *nos*, suggesting that the promoter used to drive Cas9 in the transgenic mosquito does not fully recapitulate endogenous *nos* expression levels or that, alternatively, *nos* mRNA transcripts are more stable than their Cas9 counterparts. Differences in Cas9 mRNA production (and drive capacity) between species aligns with the thought that the generation of excessive amounts of Cas9 protein is detrimental to gene-drive efficiency. Results in *A. gambiae* transgenic mosquito testes paralleled those obtained in *A. stephensi*, where we observed coincident localization and accumulation of the *nos*-Cas9 transgene and endogenous *nos* transcripts (Figure  3, Supplementary Figure S2). Both genes were localized primarily in regions corresponding to premeiotic and meiotic cells. These highly concordant accumulation patterns in the male germline in both *A. stephensi* and *A. gambiae* are consistent with the high efficiency of allelic conversion observed in males for both drives, which achieve ∼99% efficiency in both species.

### Broadening the toolbox: high-resolution transcript accumulation patterns of additional germline genes

**Figure 4:**
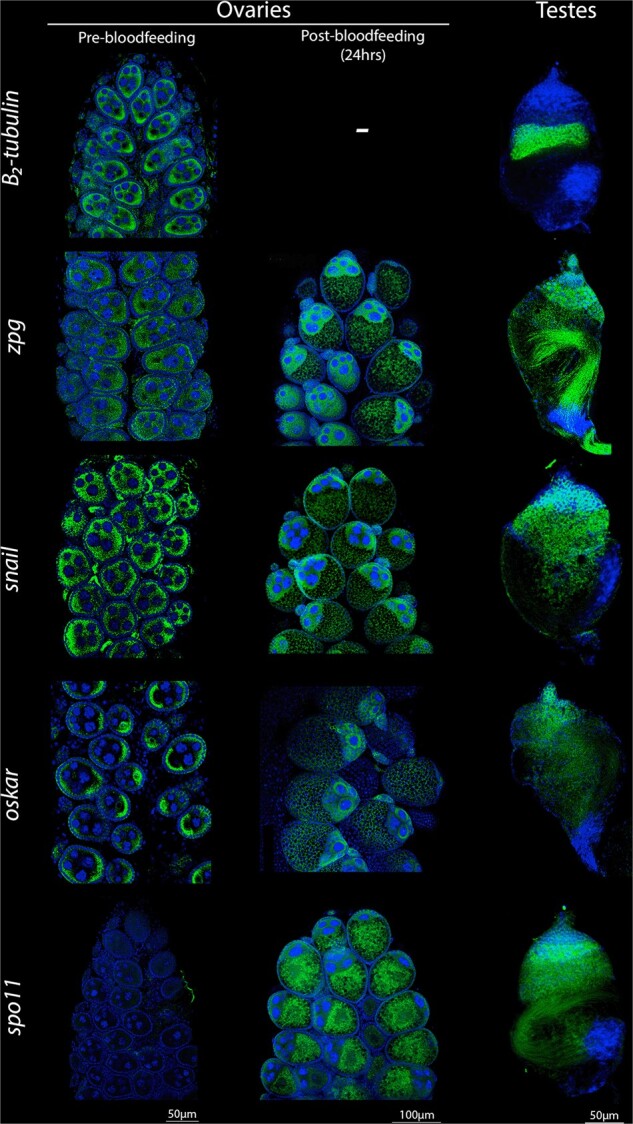
Transcription and transcript localization patterns of candidate germline genes. From top to bottom: *β_2_-tubulin*, *zpg*, *snail*, *oskar*, and *spo11.* Columns depict detection of mRNAs in ovaries pre-blood meal (5 days old), ovaries 24 h post-blood meal (8 days old), and testes (5 days old). Note: we observed cross-hybridization signals in female gonads with a similar *β-tubulin* (ASTE000064), which is expressed in both genders and shares 81% nucleotide homology to our initial target sequence, hence the detection shown in pre-bloodfeeding samples (post- was not surveyed after observing the cross-reactivity of the probe). Expression seen in testes is most likely a combination of male-specific *β_2_-tubulin* (ASTE003208) and ASTE000064.

In addition to assessing germline promoters used for the *A. stephensi* and *A. gambiae* gene drives, we also employed our *in situ* hybridization method to examine transcript accumulation profiles of other germline candidate genes known to play essential roles in the germline development through genetic studies. These genes include those whose promoters have been used in efficient gene drives such as *zero population growth* (*zpg*; Tazuke *et al.* 2002), which sustains excellent drive in *A. gambiae* (Kyrou *et al.* 2018)) and candidate genes known to play key roles in germline development. Germline developmental genes included *oskar* (Ephrussi *et al.* 1991; Kim-Ha *et al.* 1991), the transcription factor *snail* (Nieto 2002), and *spo11* (McKim and Hayashi-Hagihara 1998), a key conserved component of the meiotic recombination machinery. We examined endogenous transcript accumulation profiles for each of these genes in both male and female reproductive tissues of *A. stephensi* to evaluate whether their promoters might be of potential future interest for driving germline-restricted expression of Cas9. We used the previously described *A. stephensi* male-specific *β_2_-tubulin* gene (Yamamoto *et al.* 2019), expressed in meiotic cells of the testes, as a control of late meiotic expression and technique (Figure  4). We first confirmed that *β_2_-tubulin* transcription is indeed male-specific using quantitative gene amplification (qPCR; Supplementary Figure S3). *In situ* hybridization corroborated that the gene is highly expressed, as expected, in late stages of spermatogenesis in the testes (note that we also detected what we believe to be nonspecific cross-hybridization of this *ß_2_-tubulin* probe in female egg chambers with another tubulin homolog due to partial sequence overlap in a portion of the cDNA used to synthesize the probe—see legend of Figure  4 for details).

In the case of the *zpg* gene, transcripts were found to localize to the oocyte in pre-blood meal samples, albeit at significantly lower levels than in the testes and in contrast to the more comparable transcript levels of *vasa* or *nos* present in both male and female germlines (Figure  4). In males, *zpg* expression was observed through premeiotic and meiotic stages of development in patterns that are reminiscent of the *vasa* and *nos* genes while also generating a strong signal in the sperm flagella and vas eferens, in contrast to the other genes examined. Transcripts of developmental genes, such as *oskar* or *snail*, were localized in specific germline patterns that may render their regulatory sequences suitable for driving cas9 expression, as well (Kim-Ha *et al.* 1991; Kosman *et al.* 1991). In our studies, transcripts of both genes accumulated primarily in premeiotic regions of *A. stephensi* testes (Figure  4) similar to what we and others have observed for *vasa* (Papathanos *et al.* 2009) and *nos* (Calvo *et al.* 2005) promoter-driven Cas9 transgenes. These genes accumulated in distinct patterns in the female germline. For example, *snail* transcripts were spread throughout the follicle (Figure  4), reminiscent of Cas9 transcripts driven by the *nos* promoter in *cd:nos*-Cas9 (Figure  1D). The notable shared localization and accumulation patterns of *nos*-Cas9 and *snail* in the female germline suggest that *snail* also may sustain efficient female drive. In contrast, *oskar* accumulation mirrors its iconic pattern in the oocyte’s posterior pole (Figure  4), as previously reported both in fruit flies (Kim-Ha *et al.* 1991) and *A. gambiae* (Juhn *et al.* 2008). In the case of *spo11*, we did not observe any gonad-specific transcript localization in ovaries of nonblood fed female mosquitoes (Figure  4). Consistent with *spo11* expression being very low or absent in nonblood fed females, virtually no expression was detected by qPCR in ovaries dissected from these mosquitoes (Supplementary Figure S3). However, *spo11* transcripts were readily observed following blood feeding in nurse cells and rapidly transported into the oocyte (Figure  4). In the testes, *spo11* transcripts were comparatively more abundant (Papa *et al.* 2017; Supplementary Figure S3) and were localized in the same general spatial meiotic zone as other germline genes. These transcripts, however, appeared to be excluded from the stem cell niche at the tip of the testes that contains the germline stem cells (Figure  4).

As mentioned above, we noted striking differences in overall transcript abundance between *A. stephensi* and *A. gambiae* by qPCR. Transcripts of the former were consistently found to be at much higher levels than the latter. We examined whether these unexpected quantitative amplification differences in transcript levels were also reflected in FISH by applying the same exact confocal settings in stains for Cas9 using the same probe in both rec*kh:vas*-Cas9 and *cd:nos*-Cas9. Although FISH is not a quantitative assay due to variations that arise among samples, probe length, binding efficiency and fluorescence, the identical Cas9 probe was used for hybridization of tissue from both transgenic lines, which should detect transcripts with similar efficiency in both species. Consistent with the qPCR results, we detected much stronger signals of both *vasa* and corresponding Cas9 in *A. stephensi* compared to *nos* and Cas9 in *A. gambiae* (Supplementary Figure S4). This was further supported when we compared two stains for *oskar* using the same scanning parameters. In this case, probes were different and hence the detection could be due to differences in probe synthesis, however, the interspecies variation was very pronounced between the two (Supplementary Figure S5).

## Discussion

This study focused on the use of a high-resolution *in situ* visualization method to compare transcriptional features of two of the most-promising population modification gene-drive systems developed to date, one specific for the Asian malarial vector *A. stephensi* and one for its African counterpart, *A. gambiae*. Despite both systems being able to achieve full introduction into naïve mosquito populations, they do so based on distinct mechanisms. In particular, the *kh*:*vas*-Cas9 drive (*A. stephensi*) is copied less efficiently when transmitted via females as a result of maternally inherited Cas9/gRNA complexes generating significant rates of NHEJ alleles. The great majority of these NHEJ alleles are loss-of-function mutations that carry severe fitness costs leading to their rapid loss from the population through the transient dominantly acting process of lethal mosaicism (Guichard *et al.* 2019; Adolfi *et al.* 2020; Terradas *et al.* 2021). In contrast, the *cd*:*nos*-Cas9 (*A. gambiae*) transgene displays a highly efficient drive through both sexes, rarely generating NHEJ alleles in either gender. These differences in performance between drives motivated us to examine them at a cellular level to pinpoint where, when, and how much, Cas9 must be expressed in order to support efficient drive performance.

In the case of the *kh*:*vas*-Cas9 drive, we observed a good correlation in the localization of endogenous *vasa* and Cas9 transgene transcripts in both pre- and post-blood meal ovaries. During the arrest phase, transcripts localized around the oocyte, consistent with the general process by which nurse cells typically transcribe mRNAs and then transport them into the much less-transcriptionally active oocyte. However, 24 h after resuming transcription (triggered by the blood intake), we observed that both *vasa* and *Cas9* were present mainly around the decaying nurse cells presumably due to the difficulty in moving any transcripts into the oocyte at this stage. In ovaries, similar patterns of endogenous *vasa* distribution were reported in previous studies (Papathanos *et al.* 2009), as well as when using the green fluorescent protein (GFP) as a fusion reporter. As observed when surveying for alternative promoters, the *vasa* mRNA localization profile is recapitulated for many genes that are transcriptionally induced, or active, following the intake of a blood meal. In the testes, we observed localization of both *vasa* and Cas9 transcripts in regions furthest from the *vas eferens*. These zones correspond to stem cell and premeiotic phases of development. This interpretation of *vasa* early meiotic-specific expression is supported further by the lack of transcripts in intervening regions, where a ring of mitotically dividing stem cells resides (Supplementary Figure S1) and where the lack of expression in these supporting stem cells was also observed in a previous study using *vasa*-driven eGFP (Papathanos *et al.* 2009).

Paralleling our findings with the *kh*:*vas*-Cas9 drive, we similarly detected almost perfect correlation of endogenous *nos* and Cas9 transcripts in *cd:nos*-Cas9 transgenic testes. However, we observed discordant transcript localization in ovaries. These divergent patterns are consistent with the possibility that the *cis*-acting elements driving Cas9 expression lack subcellular mRNA localization sequences present in the endogenous *nos* transcripts. The most detailed analysis of *nos* functionality and gene architecture has been performed in *Drosophila*, where 3′UTR termination sequences play an essential role for localization and translational repression of gene transcripts (Gavis *et al.* 1996a, 1996b). One hypothesis for why the *nos* 3′UTR used to direct expression and localization of the Cas9 transgene did not recapitulate the endogenous pattern is that the transgene transcripts may lack required sequences for proper localization. The *A. gambiae* endogenous *nos* 5′UTR contains an intron close to the ATG translation initiation site that is not present in the *cd:nos*-Cas9 drive (or in other queried mosquito species), which could regulate its mRNA localization directly or interacting with its own 3′UTR similar to what occurs in *Drosophila* (Gavis *et al.* 1996a, 1996b). In support of this hypothesis, initial attempts to recapitulate *vasa* expression without introns achieved only single sex expression (Papathanos *et al.* 2009), but later proved as an excellent promoter for Cas9 expression when including them.

When comparing the design of *A. gambiae* transgenes reported in other studies (Hammond *et al.* 2021), the 5′UTR used as a promoter for Cas9 expression included this intron (Figure  1D, red box). This genome-comparable version of *nos*-Cas9, containing a full 5′UTR sequence, performed less efficiently in females than the *cd:nos*-Cas9 transgenic line analyzed in this study. While we hypothesize that the presence *vs.* absence of the intron may explain part of the divergence between the two assayed *nos* promoters, it is likely that the genomic insertion locus and gRNA target site also play a role in determining copying rates and selection for fitness. In prior experiments, the aforementioned intron was part of the construct, as well as multiple deviations from the consensus sequence that reflect natural sequence variation among mosquito populations. Perhaps, the inclusion of this small intronic fragment restricts expression of the gene in such a fashion as to limit Cas9-mediated drive efficacy, while its exclusion may lead to the imperfect overlap with the native transcript pattern. While we observed these significant differences in transcript localization, we note that *nos* transcripts are translationally repressed when not properly localized (Gavis *et al.* 1996b), and thus that the impact of improper localization of Cas9 may be reduced. Despite the imperfect overlap of transgene *vs.* endogenous transcript patterns, the transgenic *cd:nos*-Cas9 mosquito displays excellent drive performance. Whether this anomalous component of Cas9 localization influences the ability of the endonuclease to increase the frequencies of allelic conversion or, alternatively, the choice of gRNA is responsible for the high-fidelity drive remains to be determined in future experiments. In post-blood meal ovaries, endogenous *nos* transcripts localized into the oocyte, but Cas9 did not. One potential explanation for these divergent distributions is that the latter (Cas9) is excluded from the oocyte due to failure of being transported through nuclear import channels (Figure  3). This difference in post-blood meal transcript distribution may again reflect exclusion of specific *nos* upstream elements or missing terminal 3′ sequences in the *cd:nos*-Cas9 construct, but it is potentially beneficial to the drive as lack of Cas9 transcripts in the oocyte may minimize the formation NHEJ alleles as a consequence of maternal carryover to the zygote.

Complementing our analysis of the two efficient gene drives, we employed our high-resolution *in situ* detection methods to examine expression patterns of candidate genes whose germline-specific *cis*-regulatory sequences might be of potential use as efficient drivers for Cas9 in *A. stephensi*. Among these genes, *zpg* (its promoter already having been shown to be highly effective in *A. Gambiae*; Hammond *et al.* 2021) displays a pattern similar to that of *vasa*, yet at lower levels, at least in the female germline. Reduced Cas9 levels have been shown to minimize off-target and toxicity effects of the endonuclease in *Drosophila* (Champer *et al.* 2017), which may be relevant to the excellent performance of *zpg* observed in *A. gambiae* drive studies (Kyrou *et al.* 2018; Hammond *et al.* 2021). In contrast to expression in female reproductive tissues, the strong *zpg* mRNA signal detected in the sperm flagella could pose difficulties for a drive construct via the parental deposition of Cas9 into the zygote. However, previous studies did not observe this potential carryover to cause such problems (Kyrou *et al.* 2018). Another gene with a sharply delimited expression pattern is *oskar*, with transcripts localizing to the oocyte pole in nonblood fed ovaries. Given its tight transcript localization in the oocyte where germ cells form, *oskar* might prove effective in restricting Cas9 activity to meiotic components of the germline in both genders, mirroring the developmental timing in testes to premeiotic stem cells. We also observed tight meiotic localization of *spo11*, which in mammals has been used to control Cas9 expression, resulting in drive through both males (Weitzel *et al.* 2021) and females (Grunwald *et al.* 2019). In mosquitoes, *spo11* transcripts are not present prior to or during the arrest phase, but express and accumulate in the oocyte following a blood meal when transcription restarts. This observation suggests that *spo11* is either absent in early stages or that its transcripts fall below the limit of detection, possibly reflecting only very few molecules being needed to initiate recombination events during meiosis, consistent with this transcript being barely detectable in ovaries (Papa *et al.* 2017; Supplementary Figure S3). Such low expression of Cas9 should lead to minimal chromosomal damage and thus *spo11 cis-*regulatory elements may be of interest to explore in subsequent iterations of insect gene drive technologies. Strong expression of *spo11* after bloodfeeding, however, may lead to maternal deposition in the zygote and cause detrimental NHEJ events. We also noted that we detected significant difference in overall detected transcript abundance between *A. gambiae* and *A. stephensi*. Future experiments may shed light on these striking differences in apparent transcript levels, which could reflect some physical differences between the two species that limit the fraction of extractable and/or hybridizable mRNAs in *A. gambiae* relative to *A. stephensi*. Alternatively, alterations in developmental timing may exist between these fairly closely related species, which might account for some of these differences. Females were dissected at 5 days old and, while the follicle sizes were similar between the two species, transcription (or arrest) of certain genes may start later in *A. gambiae* than *A. stephensi* and thus transcripts may not be as readily detected/extracted during these stages in the former.

In summary, this study provides a novel high-resolution comparative assessment of endogenous *vs* Cas9 transgene mRNA accumulation and localization in two mosquito strains carrying high-performance gene-drive systems. Differences detected by high-resolution RNA *in situ* techniques in these two transgenic mosquito lines suggest that promoter-dependent Cas9 transcript localization may play a critical role in female drive outcomes. No appreciable disparities were detected between endogenous and Cas9 transgene RNA accumulation or localization in the testes, where transcripts were restricted to the stem cell niche and premeiotic cells for both *vasa*-Cas9 and *nos*-Cas9 systems. These initial steps toward understanding the basis for differing drive properties are important not only for elucidating Cas9-mediated cellular mechanisms but also for gene-drive developers to evaluate and predict the performance of promoters that may be used in future studies in these and other insects of potential interest.

## Data availability

Supplementary Figures S1–S6 as well as primers (Supplementary Table S1) and reagents (Supplementary Table S2) used to generate the RNA probes or perform the *in situ* hybridization experiments can be found in the main Supplementary File. Supplementary Videos S1 and S2 can be found in the main author’s public Github repository and accessed at https://github.com/GTerradas/insitu. All promoter sequences depicted in Figure  1 used to generate the *vasa* and *nos-*driven transgenic lines compared in this study (Adolfi *et al.* 2020; Carballar-Lejarazú *et al.* 2020), as well as a secondary line generated using an alternative *nos* promoter (Hammond *et al.* 2016), are available as Supplementary Data File 1 (also included in the aforementioned Github repository). *nos* 5′UTR intron discussed in the article is marked in yellow in the Supplementary file.

Supplementary material is available at *G3* online.

## Supplementary Material

jkab369_Supplementary_Figures_Tables

jkab369_Supplementary_Data
