# Supplementary material for: High-resolution in situ analysis of Cas9 germline transcript distributions in gene-drive Anopheles mosquitoes
Source: G3 (Bethesda). 2021 Nov 15;12(1):jkab369. doi: 10.1093/g3journal/jkab369 (PMC8728002; doi:10.1093/g3journal/jkab369)
Supplement: jkab369_Supplementary_Figures_Tables [file jkab369_supplementary_figures_tables.pdf]

High-resolution *in situ* analysis of Cas9 germline transcript distributions in gene-drive *Anopheles* mosquitoes

Gerard Terradas[‡1,2a], Anita Hermann[‡1,2], Anthony A. James[3,4], William McGinnis[1,2], Ethan Bier[1,2*]

[1] Section of Cell and Developmental Biology, University of California, San Diego, La Jolla, CA, 92093, USA
[2] Tata Institute for Genetics and Society, University of California, San Diego, La Jolla, CA, 92093, USA
[3] Department of Microbiology and Molecular Genetics, University of California, Irvine, CA, 92697, USA
[4] Department of Molecular Biology and Biochemistry, University of California, Irvine, CA, 92697, USA
[‡] These authors contributed equally

* Corresponding author
e-mail: ebier@ucsd.edu

Current address:
[a] Department of Entomology, Center for Infectious Disease Dynamics and the Huck Institutes of the Life Sciences, The Pennsylvania State University, University Park, PA, 16802, USA

This document contains: Supplementary Figures and Table

- Figures S1 – S5
- Tables S1, S2

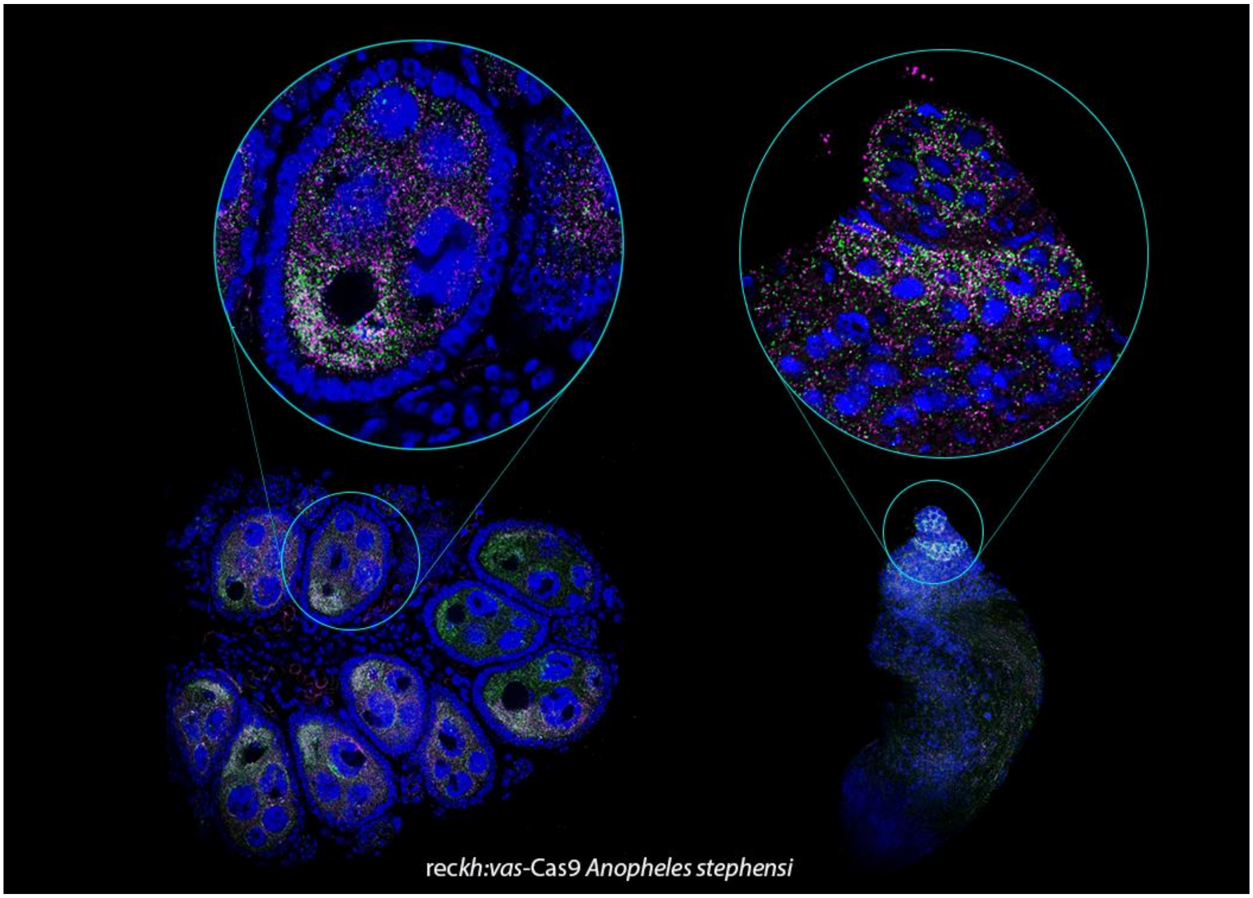

Figure S1 – High magnification images of ovarian follicle and testes stem cell niche in co-stained re*ckh:vas-Cas9 Anopheles stephensi*. A high concentration of co-localized transcripts (pearl) of the *vasa* endogenous gene (green) and *vasa*-driven Cas9 (magenta) can be observed in detail. Additional cellular compartments can be observed in higher magnification images, particularly follicle and polytene-containing nurse cells (DAPI stain, in blue) or oocyte (non-DAPI) in the female gonads and stem cell nuclei (DAPI) in males.

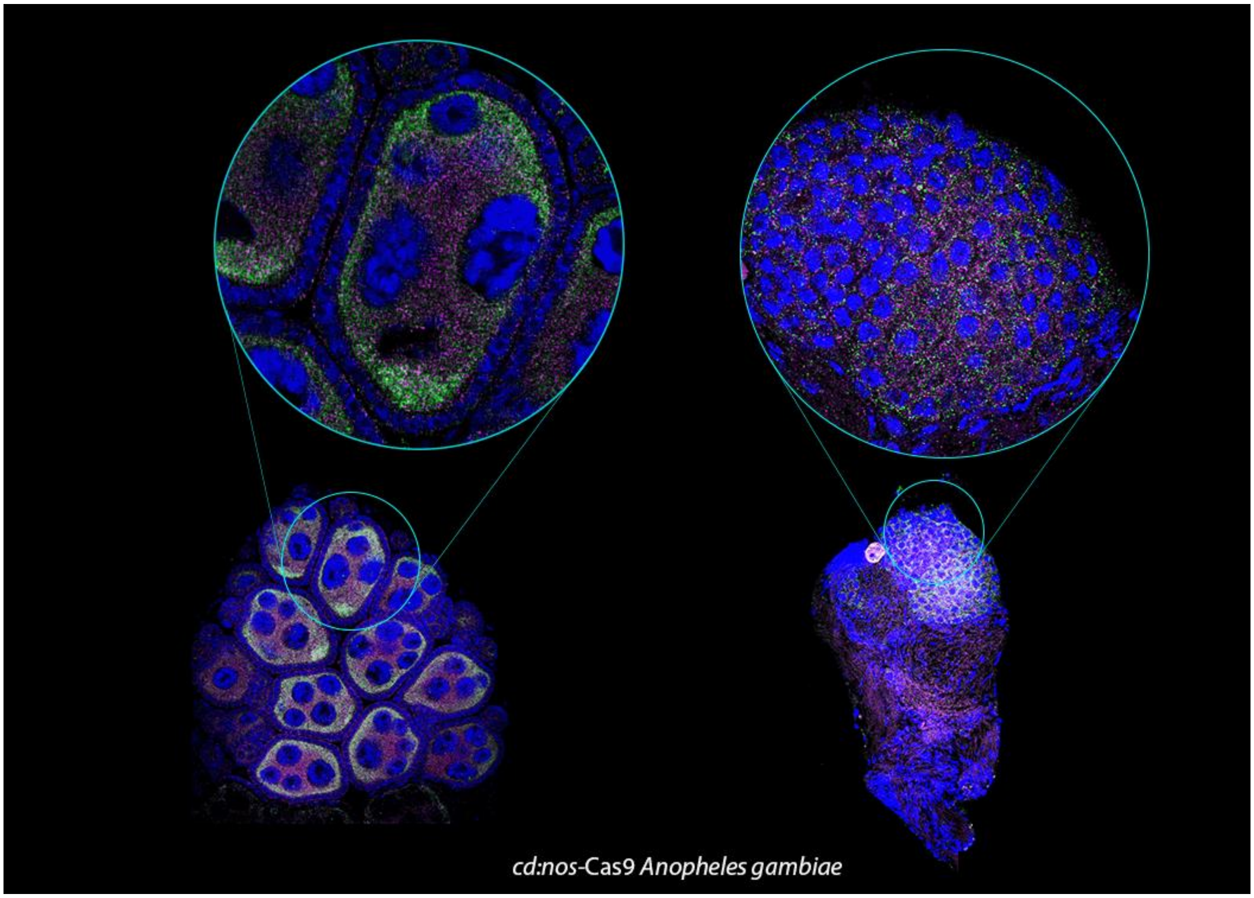

Figure S2 – High magnification images of ovarian follicle and testes stem cell niche in co-stained *cd:nos-Cas9 Anopheles gambiae*. Co-localization of single *nos* endogenous gene (green) and *nos*-driven Cas9 (magenta) transcripts can be observed in detail. Additional cellular compartments can be observed in higher magnification images, particularly follicle and polytene-containing nurse cells (DAPI stain, in blue) or oocyte (non-DAPI) in the female gonads and stem cell nuclei (DAPI) in males.

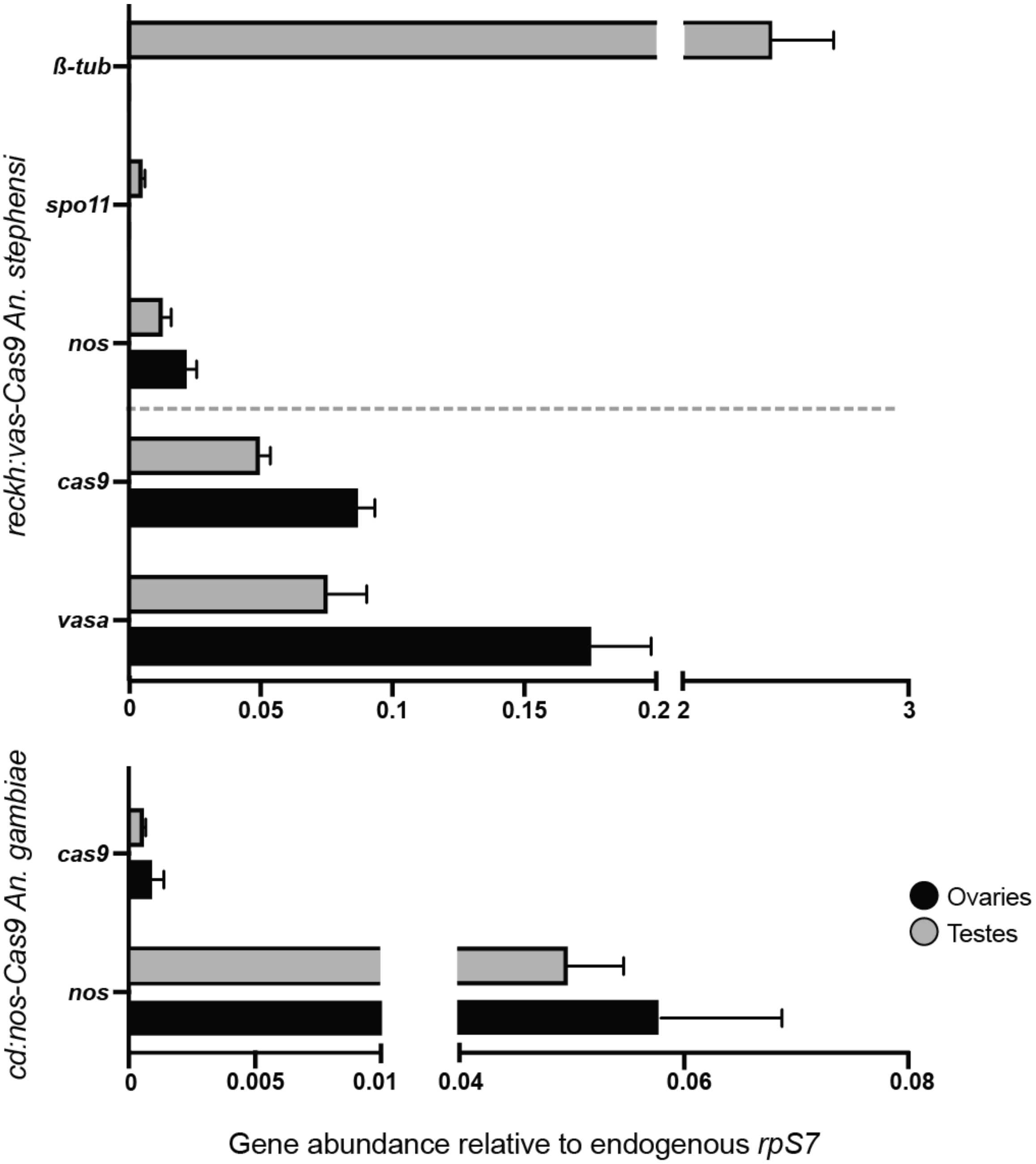

Figure S3 – Quantitative reverse transcription PCR (RT-qPCR) for the candidate genes in transgenic *An. stephensi* and *An. gambiae*. Each replicate contains approximately 40 mosquito ovaries or testes pooled to extract their RNA, which was reverse transcribed prior to performing the qPCR using gene specific primers. The mRNA abundance of each gene was normalized to species-specific rpS7 and plotted. Mean values are depicted in grey (male gonads, n=6) or black (female gonads, n=8) bars. Error bars show SD.

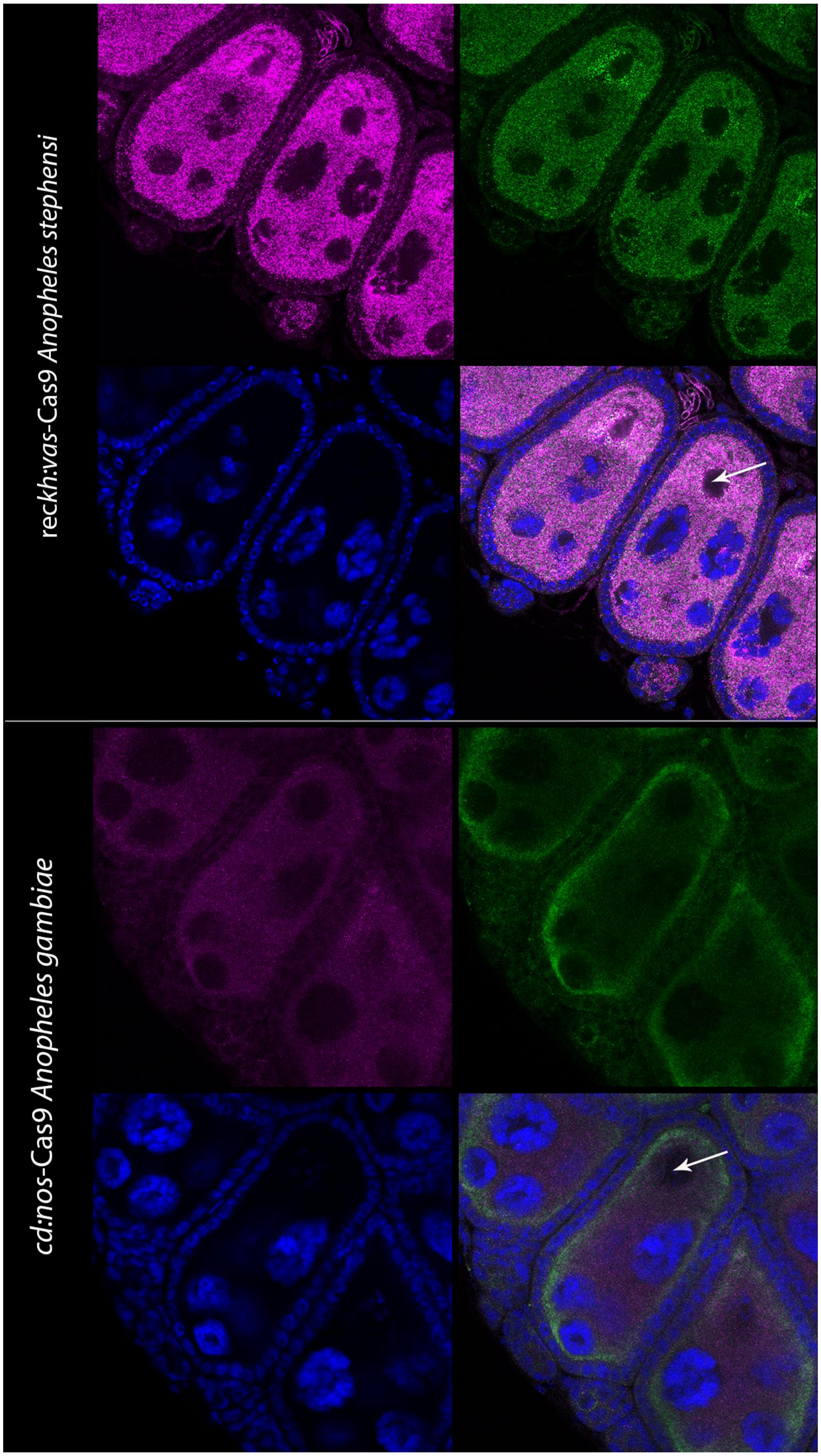

Figure S4 – Comparison between detection of transcripts in *An. stephensi* and *An. gambiae* ovarian follicles using the same exact microscopy settings. In order to confirm differences in expression obtained by qPCR, we used the exact same microscopy settings (gain, offset) to observe *in situ* stains of Cas9 from both species. Figure depicts localization of Cas9 (magenta), vasa/nos (green) and nuclei (blue) in pre-bloodmeal samples. White arrows point to the oocyte in the ovarian follicle.

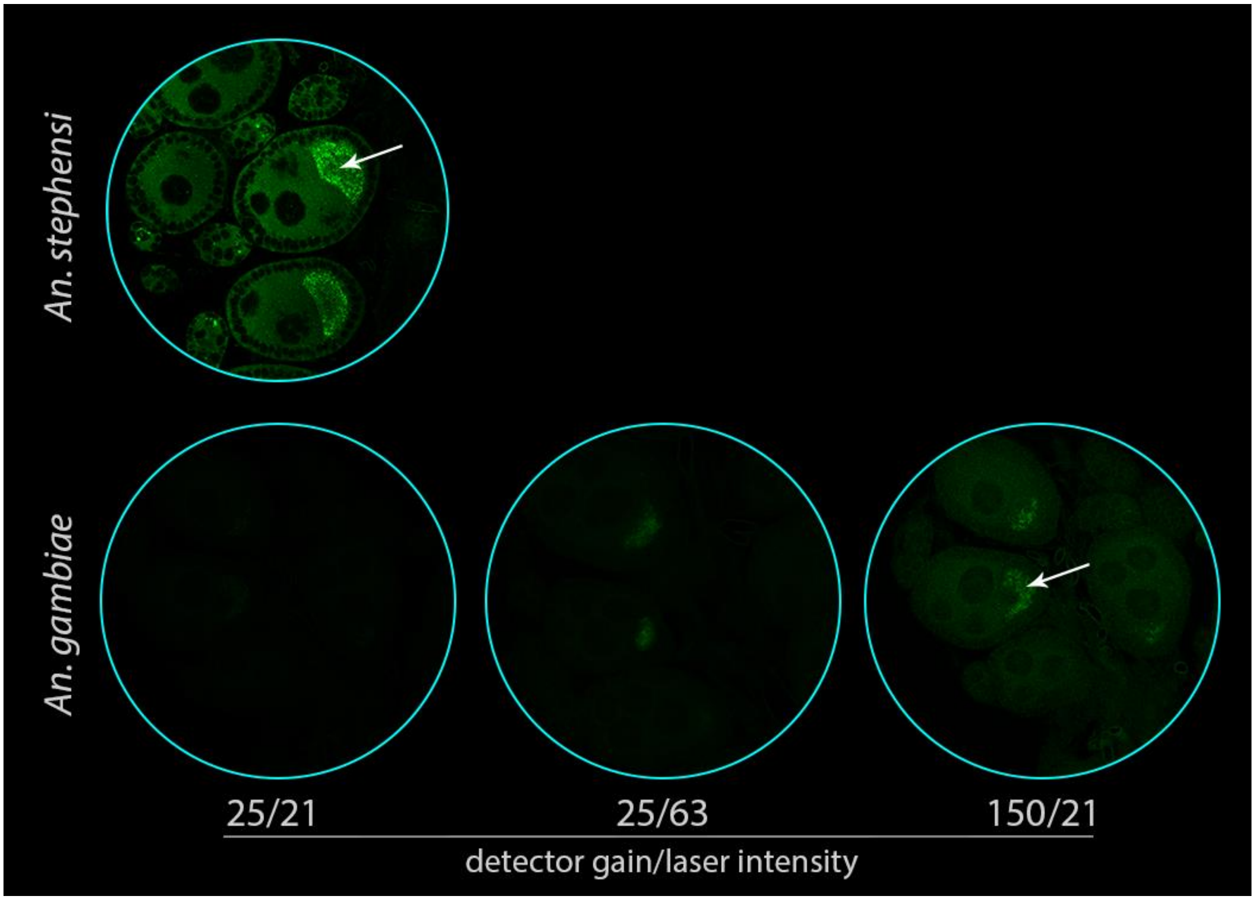

Figure S5 – Comparison between detection of transcripts in *An. stephensi* and *An. gambiae* with differing microscopy settings using probes targeting the *oskar* gene. White arrows point to the oocyte in the ovarian follicle.

Table S1 – Primers (5’ to 3’) used for different experimental assays in the paper.

	GENE ID	COMMON NAME	SEQUENCE	AMPLICON SIZE
**PROBE**	ASTE003208	Aste-B_2tub	ATGCGTGAAATCGTTCACCTACAA	1.5Kb
			TCAGTCTTCCCCGCCTTCC	
	ASTE000088	Aste-spo11	GATGGCTTCAACCAGTGCAG	2.2Kb
			TTAACCCAAGAACGGATGTTCCTTC	
	ASTE008611	Aste-snail	ATGCCAACATCGATCATGCAGAAG	1.8Kb
			CTAGTACACGGCGATGTTACCACC	
	ASTE003241	Aste-vasa	TGTGACGAGGGACGAAGCTTC	1.8Kb
			TTAGTCCCACGCTTCCTCCGG	
	ASTE002241	Aste-oskar	ATGGTTTTACTCGCACCACCCA	1.5Kb
			GTAATATCTCCACTGTTCGTCGG	
	ASTE011088	Aste-zpg	CTAGCGGTTCCAAGCAGAAC	1Kb
			CTGTGCATTATCCGCACAAC	
	AGAP006098	Agam-nos	ATGGAGGTGACGAAACAGAACG	1Kb
			CTACAAACGGACTAGCCGCTTC	
	AGAP003545	Agam-oskar	ATGGGTTTACTCGCACCAACC	2Kb
			GTAGTACCGCCACTCTTCGC	
		Cas9	CTGCTACCTGCAGGAGATCTTCTCG	3Kb
			TTCATGATGTTCGAGTAGAAGAAGTACTTGGC	
**RT-QPCR**	AGAP010592	Agam-rpS7	CCATCCTGGAGGATCTGGTA	116bp
			GATGGTGGTCTGCTGGTTCT	
	ASTE004816	Aste-rpS7	TCCTGGAGGATCTGGTGTTC	112bp
			GATGGTGGTCTGCTGGTTCT	
	ASTE003208	Aste-B_2tub	TGGATCGGACTGTTTCCTTC	122bp
			TGAATGTGTCCCTCCGTGTA	
	ASTE000088	Aste-spo11	CCGCGGTTACACTTCTTCAT	104bp
			TCGAACATTTGCTGCGTTAG	
	ASTE016369	Aste-nos	AGGATAGACCGAGACGCAAC	136bp
			GTGGCAGAACACACAATGCT	
	ASTE003241	Aste-vasa	GAGCAAACCATCCATCAGGT	105bp
			CTTCGTCTCCACAAACACCA	
	AGAP006098	Agam-nos	AGCGAACTGATGGAAGCTGT	137bp
			TCCAGCTCGCAGGTACTTTT	
		Cas9	GATGAGCACCACCAGGATCT	117bp
			ATCGATGTAACCGGCGTAAC	

Table S2 – Reagents used for different experimental assays in the paper.

	NAME IN ARTICLE	SOURCE	CAT # OR ID
**ANTIBODY**	sheep anti-digoxigenin (DIG)	Roche	11333089001
	mouse anti-fluorescein (FITC)	Roche	11426320001
	donkey anti-sheep Alexa 488	Thermo Fisher Scientific	A-11015
	donkey anti-mouse Alexa 555	Thermo Fisher Scientific	A-31570
**KIT**	RNeasy Mini	QIAGEN	74104
	TOPO TA Cloning	Thermo Fisher Scientific	K450002
**CHEMICAL**	Platinum Taq polymerase	Invitrogen	15966005
	T7 RNA polymerase	Thermo Fisher Scientific	EP0111
	SP6 RNA polymerase	Thermo Fisher Scientific	EP0131
	Ambion DNase I	Invitrogen	AM2224
	Prolong Diamond Antifade	Thermo Fisher Scientific	P36965
	DIG RNA labeling mix	Roche	11277073910
	Fluorescein RNA labeling mix	Roche	11685619910
	Formamide	Sigma-Aldrich	F9037
	Pierce 16% Formaldehyde	Thermo Fisher Scientific	28906
	iQ SYBR Green	Bio-Rad	1708880
	Salmon sperm DNA	Thermo Fisher Scientific	15632011
	Ambion Yeast tRNA	Invitrogen	AM7119
	RNAlater	Thermo Fisher Scientific	AM7021
