# Supplementary material for: High-resolution in situ analysis of Cas9 germline transcript distributions in gene-drive Anopheles mosquitoes
Source: G3 (Bethesda). 2021 Nov 15;12(1):jkab369. doi: 10.1093/g3journal/jkab369 (PMC8728002; doi:10.1093/g3journal/jkab369)
Supplement: jkab369_Supplementary_Data [file jkab369_supplementary_data.docx]

**High-resolution *in situ* analysis of Cas9 germline transcript distributions in gene-drive *Anopheles* mosquitoes**

Gerard Terradas^‡1,2a^, Anita Hermann^‡1,2^, Anthony A. James^3,4^, William McGinnis^1,2^, Ethan Bier^1,2*^

^1^ Section of Cell and Developmental Biology, University of California, San Diego, La Jolla, CA, USA

^2^ Tata Institute for Genetics and Society, University of California, San Diego, La Jolla, CA, USA

^3^ Department of Microbiology and Molecular Genetics, University of California, Irvine, CA, USA

^4^ Department of Molecular Biology and Biochemistry, University of California, Irvine, CA, USA

^‡^ These authors contributed equally

* Corresponding author

e-mail: ebier@ucsd.edu

Current address:

^a^ Department of Entomology, Center for Infectious Disease Dynamics and the Huck Institutes of the Life Sciences, The Pennsylvania State University, University Park, PA

**This document contains: Supplementary Data File.**

The following nucleotide sequences refer to promoter sequences used to generate the mosquito modification gene drives analyzed in the paper, or to the predicted sequences in Vectorbase in April 2021. Yellow-highlighted sequences show intronic 5’UTR sequence discussed in the main article file.

**Sequences for *Anopheles stephensi* gene drives *– vasa*-driven Cas9 in *kh*** (Gantz et al. 2015; Pham et al., 2019; Adolfi et al., 2020)

***vasa* 5’UTR (Gantz et al., 2015)**

ctggtaccacagtcttattggcgtgatgggttagtttaggtaatactcgacgctttataccacctagcgcgagggtcgccggcaatactcaaacctacacaaccgcgctaaagtttgcttttaactttacagctccgtttacaaatgcttaaaattatgagcttgttacaccaatgtgtgttgtttacctgtctttcgtttccggatcagcctaacgtgcagtgcctgcagcccgcactggatgagatcgctgttgatctgcaaaatgaaattaaattataatcactattctactctagtttccaacatcggaatacatacgtcagcttcagtgacctgtggggctagcccgcgaatgatgatgttattgttgggcttttgttgatggaaaaactcattgtcactgtcaaaattttccccatcatccatcgagctaggcgaatatccgccacgacggccgcccttatcgcgatccctgaaaaaaaaagcgattggcgatgaattgaatgtaacacacggcaagcaaactggtctactaccttcgatcgcggtatcgatcccgttcgcgggatctacgatcgcgatcccgttcgcgactgcgttcacgaaacctttcgcggctaaaataaattttagatagacaaatcactttaatcacgcatcccacactatgctttgctgcaatcgtacaaacctgtcccaaccgccatcatcgtacgtttgttcgttcccatcccggtacgagtcgcgttcggtgctgcgctgccggggataggagtagcgctccctgctgtacgaccgcgaccggctgcggctgtaccggctgctattgcggtaccgttccctaaaaggtttaaaaaagcaccaattaatttccggacacggtttcgtgcacacggtggggtgttccggcacaccggtgtggcggcgagacacggaacactaccactggagaccatgctacgataaggcccaaggcgagacaacggcaccagcggacggcactcttcgcaacggcgtgtctctgggtagggaggacgcatttgggcacgcatgtactccggggaggggtgctatacggtcaagcgcttacttcgatcttcctcgattgccccggtcgcgctcatcacgtcggcagcgatttctatagtagccagaagtatcactgtctggactcggtgagactataaacaatcacatttcaaaggaaattattggtcggtcgatcgaaaacatgggaagaattagggcgaacattggttcatgcaatcttcgcccgtcgctttctgtatgagggtggtctattataactttcagatataaaataactcggctgtttttttgcttttttttttgtcactaccctccagtacctatctagaacacccagccaggtagaggtgacaagatagaacataaaatgcttttgcctacagaagtggacagactgactgaatgaaggtagcctaaaacgcaaagaaaaactctcagcttttcggtggtgtacgctaggccggctggtttgttatccattcgcctttccaatcgaaccgtagcgtacacgggaaaacacacaaaaccaaaccaaaactctactacactttcaaagatggcagacttacaatccatctcggttgccttgaaacgctccagcccgaatgcgccgtctccttttgctcacggattattcacacactttccctcgattcagtgcacgtacttggaaaatgcggcttttacttaagcattgccacccttgaggacaatggactggggtaaagttattttacaattaatgtacgaagctaccaattgcacgtagaacataatcgagattttttccacaatgacaacaggcacagtgggacagccgaatggtcagctgcgttgacagtttgagtgacggttagaaagcagatcgcaatatgacatttattttcaagcaaatacgatcgcgtacaacaaaatttgttgccagaaattgaaaattattttattcaatcgaagagtttgattatcagattttaaataaagtaataaattttgtgtagctgattcgctagggaatttgtgctattgctttcgatttgctgcggtaaatcgttgcaaggctcggcacgtgttgcaaggctcggcagtggaataggatgtgtgtagagtttgtagagtgtggatcctgctatcctgctggaaactaccaaactcagtcatttcctcgaagtactcctagcgtgtaggaagcgtcgacccggtgctgttcattgctcatttcgccattgctgcgcaaccggcgctttcctgtgcttaatcaaaaccatttttgactggtaagtattatcaaggtgttttagaatcctgactactatagataaatgataaatgaaaaatatgaaaaaactatgataaatgaaaaatagctaagagtgttaaattggttggttatattggtttggttttgggtttagtagagaaaatgctatataaattttgctttcaaggattcgcttaacgatttacgcaaaacgattcattcatttcccaaaagctaagaagggtttggctgttttattgaaataatataatttccaaaattttgattcaattgtcgagcgaatcgcgccatttgccctttgtctctcggttcggtaacggtcgcgcactaacaccatcagtgcaagataaaacaaaaggtcgtggtgtgtgtgtgtgtgtgttggtgctgtcaaaatcggctgtctctgctgatgccggtacactgttgattttgcgcgtgctattcgtaaacattccttggtgcgtgctgcctcgttgtgcatgttgcgctttcgtaaaggagcaggctttcaatgtgtggtggtgcggtaggatttggagttaacttctcggaaacacgacacacacaaagcgtttggtctgtgtctccctttcctttcaaacgatccggcagcagtagcaggggatgtaggtagaactggtggcgagtagtgtagtaggtttttgcctggatagacgttatttgaggaacgaaagtggctcggtgcagttaaacccagtagtagtgtgtgcagctttgactgcagtgtgccttaccgctaccaccgagtggaagtaacaagctctgtgatatacaaagtaagaataaccgcatggtttttatcatcttcacctatcgcaagtcgttcatcggcttttggcaatcaaattagcctatatctacttcaaccaacgatcatgccgcctagtgaagaaatcatgcaccattgttgtgttccagtaaagtgcagtgcgcaatcgaatgaagttgttatttttgctagggctagtgttactactggctggcctctatcaggggtctcgaaatgcgcacgaaatgcctctccgtgcacgttccgggacggcggatgagcatatcgttgtcgttcatccgcaaataattgatgcgaaaagcgcgcgcggtccggcacatcgtctctgctagcggtatatctctctaccctcacagtcacacgcgcccaccgtccataggctacttcgtcgtgtagtgtagtggtggtggtcgcctggatgaaagcagtattgtcgcttttaaaattttcttgggaaaagcttttgtggctctgtgtgttgcccccttgcctgtgaaacgatcgtttgtgccggcggcaaagttgttggtatttgtatgtgtgtgtgattttctttacagcacacaggagtgactgcttgtagtaatagttcgtccatttttttctgtatctttcttccatgcacgtgcacacaccgtcacttttaccggtagctgcttgctgatcgccattacgaatcgaagagagagaagagagggtcgtcacgccgttagttactctacactaaactgctgccccgcttacggacgacttgagcctgcgtgatctttctacagaatcgttacgcatttggaggaaatttgcagcccgacaccatcacttcagcggcgtgcttaaaatcgatctaggaag

***vasa* 3’UTR (Gantz et al., 2015)**

cgagagttcggtgcgaatctctctcttgatttttcccgttttttttccttataattacaacattagcgaaacttcagcgcgagtaagtttcgtaaacaaaagagctagagtaaaactctctatattccctcgtatagcgaagtgcgtgatgatttttctacatttaatgacactatgtactaaaaaaaaagcatggcgcagcggggctgttgaataaatggttgttagcccctcaggggtggaacttgagatcgcttttcgtggttttgcactacaggtagattgaaaaatcagtaatccgaacatagtattgtcaacttttggagcttgacgtatacccgcttaaaagctataacactccagcgacggaagtggacacgaaggctgtcgagtagtcgggaggaggatgtaagagtcaccaagcctgtccatagaagtagtagtggggagattgcacgctaagttgaatcggaaatccggtggtccttaaacctcaggaacgattaaaggcatgaagcgatgtctttcatgaaagtctcacatcaggaataactaaattcgaccttacgagaagagggactgaagcggtgactgactactaagaagctgaagtcgatgggaaagacaccagagaaggcgagaattcttaggcgactatatctagggaaaatctctaagctacgaaaaatcgtataagcttttacttgatcataagtacttttattgatagttcgctatagctccggtcgacataacaagcacagcattagcttaattcgcgcagatatcatcggcctgggatgaagcatcctgcaacgactgcaacatcagcgtgcgtgcattcgtcagacactcctcaaaggctgacttcaaaaccccggacaattcgccggtcacaccggttccctctatggcgactggattgctcaacttctcctcgatccttgcctgaatcgtgaacggctcaacgaagatgttcacatcccgcaagctgctcaacaggctggccttggt

**Sequences for *Anopheles gambiae* gene drives *– nos*-driven Cas9 in *cardinal*** (Carballar et al. 2020) **or *doublesex*** (Hammond et al., 2016; Hammond et al., 2021)

***nos* 5’UTR (Carballar et al., 2020)**

ggccgtgtggtttaacccaccaaactggcagtttcttggctttgttcgatgcaactggatttttagtacaggaaattggtggcgttttcggtatcttggttatttgggtagtgtatagtggtgttttcgaaattcatttttgttcattaacagttgttcaacctatagttatttattacaataatattctactaacgattaaccgattgattcaaagtgaataaattataaaactagtgattttttttTaaatttttgtatgattttgtcatttcttggatcattatccgtgcaaatcgattaaccggcaacagtccggtcccgagctgcccgaataatcgacgttctactgtattcctaccgactttttgatatgcctactgactcaccgatgagccccctaaaactaccgattttttatttatcctaccgaaaatcacagattttttcgtaatactgaccaaaaagtcatgtaatcatttcccaaatcacttaatgtattaaactccatatggaaatcactagcaaccagaacaagaagttcaacagagacaacccccgtgtacttcatgagatgcgatgatatcggacgcgctggtaaaattttatatggtatttgaaagatactgtaagacgtgcgattttttaatacgatggaatcaattcaacagtcaattatgaattaattaagaatcaatttgtagaaaaaatctctaatgtatgttttgttatcgtaacagttaccaattcatccaaataatcgacatagcgacacagcacattcgatttagcaatgtcctaagcggccgcgtggagccccaaaaaaagggagtccgcaacgcttagaagcaaatgtgcatctcaatcattcgttgggttagaatccctagtacaattttcagattgacacttcagaaaggcctcatgtgactgctatgaaccaaatataaactatcacctttaaatagataatggatgtattttgtcgtgctactgaactttcgttgggaaaattaggggtagaacccccccccccccccgttcaacacttctaaacctaccgataaaatttgggtgcgcctactgaaaaccgccaaaataatctggccacactggctgcatttgatgttttgaaacatcaccaaattttactagatactgcacttgagcgtcgttgaagctgaactacagctgaattgcgctttcttaaatattaccaacttggtatttaattttttttatatacttcaatctaaaactgtttcttggaatgataattttacccaaaaccaaaatcacaaagagtgtgaggttggtggtcccgccggctactgcaacttgtggccatcgctcatctcacgcacacatatgcacacatctgtcatttgaaaagccgcacacaatcgtgtgttgtgcaaaaaaccgttcgcgcacaaacagttcgcacatgtttgcaagccgtgcagcaaagggtttctgatggtgatccgcagtgtttggtcagctttttaatgtgttttcgcttaatcgcttttgtttgtgtaatgttttgtcggtataatttttatgcattgttacaaatgaaatgtataatcctgcgttactagtgtaaaacattgctaattcccgtctagaaagcaag

***nos* 5’UTR (Hammond et al., 2016)**

gtagtgtctagtggtattttcgaaattcatttttgttcattaacagttgttaaacctatagttattgattaaaataatattctactaacgattaaccgatggattcaaagtgaataaattatgaaactagtgatttttttaaatttttatatgaatttgacatttcttggaccattatcatcttggtctcgagctgcccgaataatcgacgttctactgtattcctaccgattttttatatgcctaccgacacacaggtgggccccctaaaactaccgatttttaatttatcctaccgaaaatcacagattgtttcataatacagaccaaaaagtcatgtaaccatttcccaaatcacttaatgtattaaactccatatggaaatcgctagcaaccagaaccagaagttcaacagagacaaccaatttccgtgtatgtacttcatgagatgagattggacgcgctggtaaaattttatatgggatttgacagataatgtaaggcgtgcgatttttttcatacgatggaatcaattcaagagtcaattgtgcaggatttatagaaacaatctcttatttatgttttgttatcgttacagttacagccctgtcctaagcggccgcgtgaaggcccaaaaaaaagggagtccccaacgctcagtagcaaatgtgcttctctatcattcgttgggttagaaaagcctcatgtgacttctatgaacaaaatctaaactatctcctttaaatagagaatggatgtattttttcgtgccactgaactttcgttgggaagattagatacctctccctccccccccctccctttcaacacttcaaaacctaccgaaaactaccgatacaatttgatgtacctaccgaagaccgccaaaataatctggccacactggctagatctgatgttttgaaacatcgccaaattttactaaataatgcacttgcgcgttggtgaagctgcacttaaacagattagttgaattacgctttctgaaatgtttttattaaacacttgttttttttaatacttcaatttaaagctacttcttggaatgataattctacccaaaaccaaaaccactttacaaagagtgtgtggttggtgatcgcgccggctactgcgacctgtggtcatcgctcatctcacgcacacatacgcacacatctgtcatttgaaaagctgcacacaatcgtgtgttgtgcaaaaaaccgttcgcgcacaaacagttcgcacatgtttgcaagccgtgcagcaaagggcttttgatggtgatccgcagtgtttggtcagctttttaatgtgttttcgcttaatcgcttttgtttgtgtaatgttttgtcggaataatttttatgcgtcgttacaaatgaaatgtacaatcctgcgatgctagtgtaaaacattgctaattcccggtaagaacgttcattacgctcggatatcatcttacgaagcgtgtgtatgtgcgctagtacattgacctttaaagtgatccttttgttctagaaagcaag

***nos* predicted 5’UTR (Vectorbase)**

CGCGCACAAACAGTTCGCACATGTTTGCAAGCCGTGCAGCAAAGGGTTTCTGATGGTGATCCGCAGTGTTTGGTCAGCTTTTTAATGTGTTTTCGCTTAATCGCTTTTGTTTGTGTAATGTTTTGTCGGTATAATTTTTATGCATTGTTACAAATGAAATGTATAATCCTGCGTTACTAGTGTAAAACATTGCTAATTCCCGGTAAGAACGCTCGGATATCGTCCTACAAGGCGTGTGTATGTGCGTTAGTACGTTAACCTTCTAAAGTGATCCTTTTGTTCTAGAAAGCAAG

***nos* 3’UTR (Carballar et al., 2020)**

Gacagagtcgttcgttcattccttttttattactttacaacacatccaaagctctgtgagcttcaagcaacaggtagtagctgacatcggaactggtgggcaagaaaggcttgcagcaaatacgtttttggctgctcagagaatgtgaagcttgaagatatatttatttaggaaaagtggaactttatgcaggatgaataattttgccatcgaatcaaatagcgtaagtaggtagagtgaaaaatcgatcttaaaaggatgatttccacgttcgaacattacacattaaggatggtatccatacatacgaatgcggtttaaattcaatatttaccttgaagcagatgttcgtatcatttcctccttagcatctttatgtctatacttcttttaaggacaacatttatagattttttgatacaacgaatcattttctatgaatcaatcatttgatgcattgattaaataaattgcgaagaaatatttaacaacaatcgattctaaatgtgttgttagaaggtacaaccaaaaaacctttaatgtcttggagcgaatgttcaaagatattgcttagcctctctcttcggctaaaatgaacactaattac

***nos* 3’UTR (Hammond et al., 2016)**

gacagagtcgttcgttcattccttttttattactttacaacacatccaaagctctgtgagcttcaagcaacaggtagtagctgacatcggaactggtgggcaagaaaggctttcagcaaatatgtttcaggctgctcggagaatgttgaagatatatttatttaggaaaagtggaactttatgcaggatgaataattttgccatcgaatcaaatagcgtaagtaggtagagtgaaaaattgatcttaaaaggatgatttccacgttcgaacattacacattaaggatggtatccatacatacgaatgcggtttaaattcaatatttaccttgaagcaggtgttcgtatcatttcctccttagcatctttatgtctaaacttctttcaatgacaacatttatcgattttttgatacaacgaatcattttctatgaatcaatcacttgatgccttgattaaataaattgcgaagaaatatttaacaacaatcgattctaaatgtgttgttagaagatacaaccaaaaaacctttaatatcttggagcgaatgttcaaagatattgtttagcctctctcttcggctaaaatgaacactaattac

***nos* predicted 3’UTR (Vectorbase)**

GACAGAGTCGTTCGTTCATTCCTTTTTTATTACTTTACAACACATCCAAAGCTCTGTGAGCTTCAAGCAACAGGTAGTAGCTGACATCGGAACTGGTGGGCAAGAAAGGCTTGCAGCAAATATGTTTTTGGCTGCTCGGAGAATGTTGAA
